# Perioperative complications of en bloc resection and anterior column reconstruction for thoracic and lumbar spinal tumors

**DOI:** 10.1186/s12891-024-07408-y

**Published:** 2024-05-09

**Authors:** Yanchao Tang, Haozheng Li, Shanshan Liu, Jiacheng Liu, Hua Zhou, Xiaoguang Liu, Zhongjun Liu, Feng Wei

**Affiliations:** 1 Department of Orthopaedics, Peking University Third Hospital, Beijing, China; 2 Beijing Key Laboratory of Spinal Disease Research and Engineering, Beijing, China; 3 Research Center of Bone and Joint Precision Medicine, Ministry of Education, Beijing, China; 4 Health Science Center, Peking University, Beijing, China

**Keywords:** Spinal tumor, Thoracic and lumbar spine, En bloc resection, Anterior column reconstruction, Perioperative complication

## Abstract

**Purpose:**

To evaluate the perioperative clinical outcomes of en bloc resection and anterior column reconstruction for thoracolumbar spinal tumors.

**Methods:**

This study conducted a retrospective analysis of prospective data collection of 86 consecutive patients, including 40 males and 46 females, with an average age of 39 years (ranged from 10 to 71 years). There were 35 cases of a malignant primary tumor,42 cases of an aggressive benign tumor, and nine cases of metastases. The main lesions were located in 65 cases of thoracic spine, 17 cases of lumbar spine, and 4 cases of thoracolumbar spine. Tumors involved one level in 45 patients, two levels in 12 patients, three levels in 21 patients, four levels in five patients, five levels in two patients, and six levels in one patient.

**Results:**

According to the Weinstein-Boriani-Biagini surgical staging system, all patients achieved en bloc resections, including 74 cases of total en bloc spondylectomy and 12 cases of sagittal resections. The mean surgical time was 559 min (210–1208 min), and the mean total blood loss was 1528 ml (260–5500 ml). A total of 122 complications were observed in 62(72.1%) patients, of which 18(20.9%) patients had 25 major complications and one patient (1.2%) died of complications. The combined approach (*P* = 0.002), total blood loss (*P* = 0.003), staged surgery (*P* = 0.004), previous surgical history (*P* = 0.045), the number of involved vertebrae (*P* = 0.021) and lumbar location (*P* = 0.012) were statistically significant risk factors for major complication. When all above risk factors were incorporated in multivariate analysis, only the combined approach (*P* = 0.052) still remained significant.

**Conclusions:**

En bloc resection and anterior column reconstruction is accompanied by a high incidence of complications, especially when a combined approach is necessary.

## Introduction

En bloc resection in the spine is a procedure of surgical oncology aimed at completely removing a tumoral mass, making it thoroughly surrounded by a continuous layer of healthy tissue, which is indicated for primary malignant tumors, aggressive benign tumors and infrequently solitary metastatic lesions. It can reduce intraoperative tumor contamination, thereby improving local and systemic prognosis [1–3]. However, if the vertebral body needs to be removed, the risk of complications is high, such as cerebrospinal fluid leakage, pleural effusion, vascular injury, neurological injury, and perioperative mortality rate [4, 5], which is an important deterrent for surgeons. Given the rarity of eligible spine tumors, there has been few reports of perioperative clinical results of this technique.

This study is prompted to investigate the feasibility and perioperative complications of en bloc resection and anterior column reconstruction for thoracic and lumbar tumors by reviewing 86 consecutive cases treated in our institution.

## Methods and materials

### Study patients

This was a retrospective study of prospectively collected data of 86 consecutive patients who underwent en bloc resection and anterior reconstruction for thoracic and lumbar tumors at our hospital from May 2016 to October 2022. There were 46 females and 40 males, with a mean age of 38.8 years (range, 10–71 years). The most common tumor types were giant cell tumor (38 cases, 44.2%) and chondrosarcoma (11 cases, 12.8%). There were 35 cases of a malignant primary tumor, 42 cases of an aggressive benign tumor, and nine cases of metastases (Table 1). Enneking classification grades of primary tumors were S3 (42 cases), IA (one case), IB (14 cases), IIB (19 cases) and III (one case) [6]. Tomita scores of metastases were 2 in two cases, 3 in two cases, 4 in one case and 5 in four cases [7]. The main lesion was located in the thoracic spine in 56 patients, in the lumbar spine in 26 patients, and in the thoracolumbar spine in four patients. Tumors involved one level in 45 patients, two levels in 12 patients, three levels in 21 patients, four levels in five patients, five levels in two patients, and six levels in one patient. There were 22 patients with recurrent tumors.

**Table 1:** Pathological classification

Pathology	Number	Frequency
Giant cell tumor	38	44.2%
Chondrosarcoma	11	12.8%
Chordoma	6	7%
Leiomyosarcoma	5	5.8%
Osteoblastoma	4	4.7%
Osteosarcoma	3	3.5%
Chondroblastoma	2	2.3%
renal cell carcinoma	2	2.3%
Solitary fibrous tumor	1	1.2%
Solitary fibroadenoma	1	1.2%
Malignant transformation of GCT	1	1.2%
Rhabdomyosarcoma	1	1.2%
Phosphaturia mesenchymal tumor	1	1.2%
Malignant perivascular cell tumor of the meninges	1	1.2%
Embryonal rhabdomyosarcoma	1	1.2%
Schwannoma	1	1.2%
Spindle cell sarcoma	1	1.2%
Angiosarcoma	1	1.2%

### Treatment decision making

Diagnosis of non-recurrent cases was made based on histopathology reports of core needle biopsy. Computed tomography (CT), magnetic resonance imaging (MRI), X-ray radiograph of the spine and positron emission tomography (PET-CT) were performed on all patients. Strategy of treatment for each patient was determined by the same multidisciplinary team of surgeons, pathologists, radiologists, radiotherapists, chemotherapists and anesthesiologists. Each time after the surgical plan was made, the patient was thoroughly briefed regarding the procedure and the morbidity associated with it. Preoperative angiography and embolization were recommended to all cases. If preoperative radiotherapy would be required, depending on tumor histology, the patients underwent surgery after 30 days but no later than 40 days after radiotherapy.

### Surgical procedure

En bloc resection was planned by the guidance of the Weinstein-Boriani-Biagini (WBB) surgical staging system, considering location and size of the tumor and involvement of surrounding neurovascular structures, as evident on radiographic studies [8]. The surgical procedure required release of surrounding neurovascular structures from the tumor, en bloc resection of the tumor, reconstruction of the anterior defect and instrumentation. We would seek help from another specialist surgeon in the following cases: the tumor was closely related to great vessels, a transperitoneal approach was needed, or if other affected organs needed to be removed simultaneously.

**Fig. 1:**
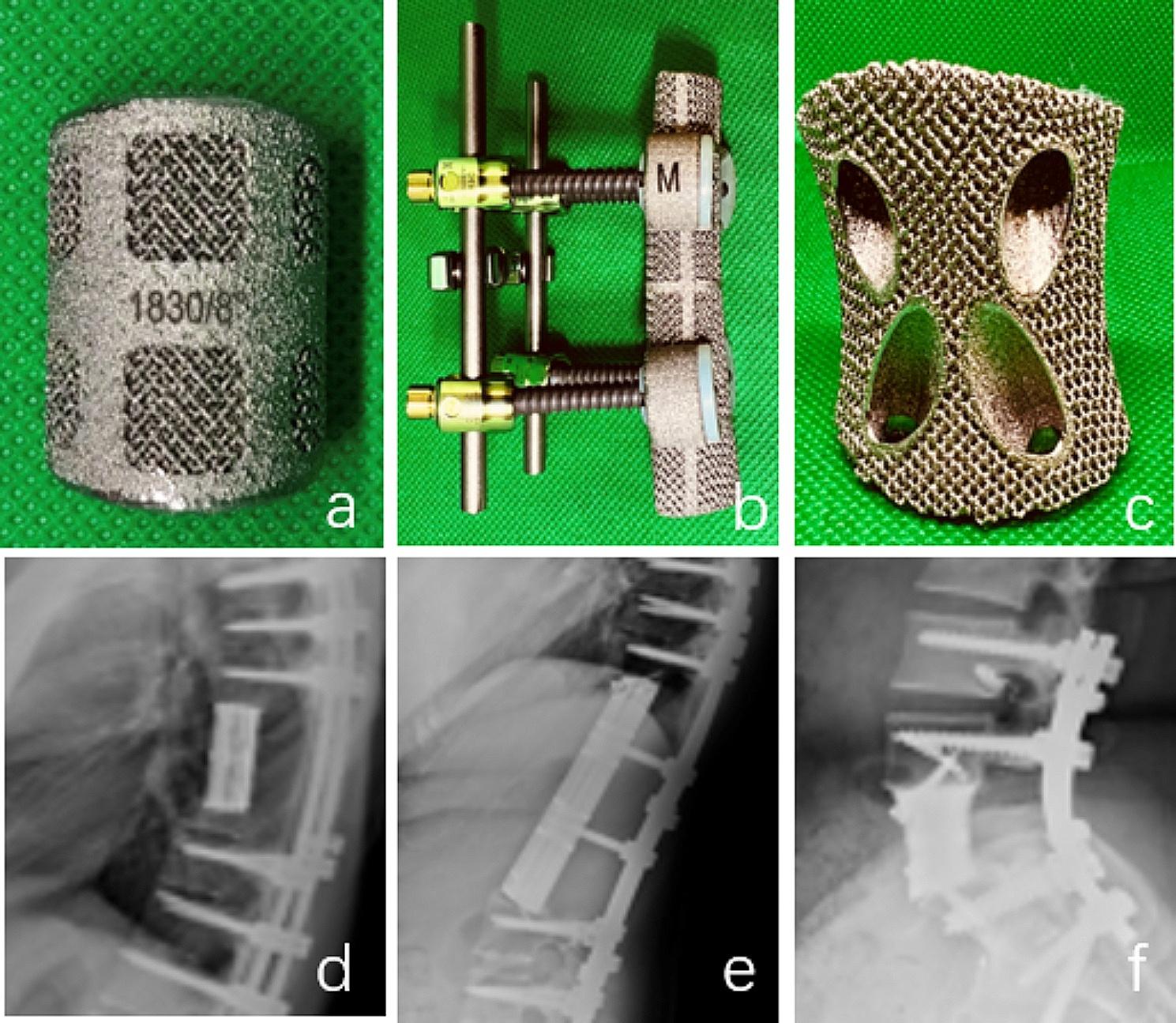
(**a**) Standard 3D-printed vertebral body. (**b**) Truss structure 3D-printed vertebral body. (**c**) Anterior self-stabilizing 3D-printed vertebral body-connection to vertebral body. (**d**) X-ray of standard 3D-printed vertebral body after surgery. (**e**) X-ray of truss structure 3D-printed vertebral body after surgery. (**f**) X-ray of Anterior self-stabilizing 3D-printed vertebral body after surgery

For the majority of thoracic tumors or one-level upper lumbar tumors, a single posterior approach was sufficient if the anterior visceral and vascular structures were not involved. The surgery required removal of all posterior structures, bilateral costotransversectomy in the thoracic spine, and thorough release of the spinal cord or cauda equina. In case of pedicle involvement, the pedicles were left in situ and removed en bloc with the tumor. A plane was created between the anterior vascular structures and the anterior wall of vertebral body. Malleable retractors were placed to protect the anterior structures. The caudad and cephalad discs or vertebral bodies were then removed and the tumor, along with the vertebral bodies, was removed en bloc in a rotating maneuver from posteriorly. In patients who had sagittal resection, the posterior vertebral osteotomy was done using an ultrasonic osteotome and osteotomes while maintaining anterior control and protection of the major surrounding organs with malleable retractors. Once the tumor was resected, it was sent for X-ray and CT scan to confirm the margins achieved. The caudad and cephalad end plates or vertebral bodies were then curetted to prepare a vascular bed. Followed by reconstruction of the anterior bone defect using a 3D-printed vertebral body. Rods were then applied and compressed. We also used pedicle screws to connect the vertebral body through prefabricated holes anteriorly and rod posteriorly (Fig. 1). We hope this would protect the spinal cord and provide additional stability. Thorough wash using normal saline and dilute iodophor solution was given, drains were placed and skin closed in layers.

If the tumor broke through the anterior cortex of the vertebral body, anterior dissection was required, followed by posterior resection and reconstruction. In anterior–posterior surgeries, once release was completed, a gauze or rubber pad was placed anteriorly to the tumor, protecting the neurovascular structures and surrounding organs, which would later be accessible and removed from the second posterior approach. After completing the anterior release, the patient was positioned prone for the posterior en bloc resection similar to the steps described above.

If the paravertebral tumor was too large to be removed posteriorly, or the posterior removal might cause injury to lumbar roots, especially if the tumor is located in the L4 or L5 vertebral body, resection of the posterior structure was required, followed by tumor removal and reconstruction anteriorly. In this case, the function of lumbar roots, especially the walking function, can be preserved. In two three-level lumbar tumor cases, we also went posterior–anterior–posterior to apply a posterior compression to stabilize the prostheses.

A single anterior resection and reconstruction was carried out if the tumor was confined to the vertebral body and did not involve the pedicles bilaterally. In general, the choice of approach depended on the margin of the tumor and its relationship with extra-vertebral vital structures.

### Aftercare

Postoperatively, patients were transferred to the intensive care unit (ICU). Drains were removed once the collection was less than 50mL. Patients were started on deep vein thrombosis prophylaxis (low-molecular-weight heparin) the next morning and calf pumps for one week. Mobilization was started with the protection of brace as soon as possible. Stereotactic radiotherapy and/or adjuvant chemotherapy was initiated at week four postoperatively based on postoperative histopathologic results.

### Data collection and outcome measures

Patient demographics, diagnostic and treatment profiles, morbidity and mortality outcomes were reviewed. Perioperative complications were divided into major and minor as described by McDonnell et al.: major complications were considered as any complication that appeared to substantially alter an otherwise full and expected course of recovery, and other complications were regarded as minor [9]. In particular, neurological decline of more than one grade, as assessed by the Frankel score, was considered a major complication [10]. Resected tumor data were studied with respect to histopathology margins. Type of excision was classified into intralesional, marginal, and wide margins (Table 2).

**Table 2:** Type of excision

Intralesional	if the tumor was violated, thereby causing tumor spillage.
Marginal	if a thin shell of normal tissue covered the tumor without breach of tumor tissue through the shell.
Wide	if a thick layer of peripheral healthy tissue, a dense fibrous cover (e.g., fascia), or an anatomic barrier not yet infiltrated (e.g., pleura), fully covered the tumor.

### Statistical analyses

Continuous variables were expressed as mean ± standard deviation, if Gaussian, or as median and 25th -75th percentile, if skewed. Normality of distribution was assessed by means of the Kolmogorov-Smirnov test. Categorical data were shown as absolute and relative frequencies.

The student t test and the chi-square test were used to compare the surgical outcomes of patients receiving initial or revision surgeries. A logistic regression analysis was applied to find predictors of major complications, considering demographic, oncologic and surgical parameters as covariates. The multivariate model included only covariates with a *P* < 0.10 in univariate analysis. Calibration of the multivariate model was evaluated by the Hosmer-Lemeshow test. Logistic regression analyses were then applied to find predictors of neurological deterioration, one of the most common major complications, and of minor complications in patients who had not experienced major complications.

A 2-sided *P* value < 0.05 was considered to be significant. For all analyses, SPSS 26 statistical software was used (SPSS Inc., Chicago, Illinois, USA).

### Ethics

All procedures performed in this study involving human participants were in accordance with the ethical standards of the institutional research committee and with the 1964 Helsinki declaration and its later amendments or comparable ethical standards. Informed consent was obtained from all individual participants included in this study.

## Results

There were 49(57%) patients who received neoadjuvant therapies (chemotherapy/radiotherapy/targeted therapy). En bloc resection was achieved in all patients, including total en bloc spondylectomy in 74 cases, sagittal resection in 12 cases. The operative approach was performed as a single anterior approach in one case, a single posterior approach in 42 cases, an anterior-posterior approach in 28 cases, a posterior-anterior approach in 14 cases, and a posterior-anterior-posterior approach in two cases. Seventy-three patients went through all procedures at the same day and 13 patients underwent the second procedure at a different date for safety concerns raised by prolonged surgical time or hemodynamic instability. The mean operating time was 558.6 min (210–1208 min). There were 66 (76.7%) patients who underwent a preoperative embolization and the mean total blood loss (sum of the blood loss of staged operations) was 1527.6 mL (range, 260–5500 mL). The mean stay in the hospital after the operation was 14.0 days (4–88 days). Postoperative CT scans and histopathologic analyses indicated that wide margin was achieved in 42 (48.8%) patients, marginal margin was achieved in 16 (18.6%) patients, and intralesional margin was achieved in 28 (32.6%) patients who subsequently underwent postoperative radiotherapy.

A total of 122 complications were observed in 62 (72.1%) patients, including one major complication in 11 (12.8%) patients, two major complications in six (7.0%) patients, three major complications in one (1.2%) patient and 96 minor complications in 55 (64.0%) patients. The neurological status of 10 (11.6%) patients decreased by more than one grade of the Frankel score. There were five (5.8%) major infections requiring debridement and antibiotics for 6 weeks. Three (3.5%) patients had great vessel injury. Two (2.3%) patients required adjustment of instrumentation. One (1.2%) patient had ureteral injury, one (1.2%) patient had recurrent laryngeal nerve injury, one (1.2%) patient had lung injury and one (1.2%) patient underwent tracheotomy because of weak expectoration respectively. One patient (1.2%) died of complications because of cardiopulmonary arrest. (Table 3)

**Table 3:** Overall perioperative complications

Type of complication	Total	Major	Minor
Death	1(1.2%)	1(1.2%)	0(0%)
Neurological	22(25.6%)	11(12.8%)	11(12.8%)
Infection	10(11.6%)	5(5.8%)	5(5.8%)
Vascular injury	3(3.5%)	3(3.5%)	0(0%)
Cardiopulmonary	6(7.0%)	3(3.5%)	3(3.5%)
Hardware related	4(4.7%)	2(2.3%)	2(2.3%)
Ureteral injury	1(1.2%)	1(1.2%)	0(0%)
Pleural effusion	35(40.7%)	0(0%)	35(40.7%)
Cerebrospinal fluid leakage	24(27.9%)	0(0%)	24(27.9%)
Chylous leakage	5(5.8%)	0(0%)	5(5.8%)
Hematological	4(4.7%)	0(0%)	4(4.7%)
Thrombosis	3(3.5%)	0(0%)	3(3.5%)
Cerebral hemorrhage	2(2.3%)	0(0%)	2(2.3%)
Delirium	1(1.2%)	0(0%)	1(1.2%)
Intestinal obstruction	1(1.2%)	0(0%)	1(1.2%)
Total	122	26	96

Patients who underwent revision surgery were more likely to have multilevel involvement (18/22 vs. 23/64, *P* = 0.000) and major complications (8/22 vs. 10/64, *P* = 0.040) than those who underwent initial surgery (Table 4). The combined approach (odds ratio [OR] = 25.815, *P* = 0.002), total blood loss (*OR* = 1.001, *P* = 0.003), staged surgery (*OR* = 6.576, *P* = 0.004), previous surgical history (*OR* = 3.086, *P* = 0.045), number of involved vertebrae (*OR =* 1.652, *P* = 0.021) and lumbar location (*OR* = 3.509, *P* = 0.012) were statistically significant risk factors for major complication occurrence. For the prediction of a major event to occur, multivariate analysis was performed. In this analysis, the combined approach (*OR* = 9.236, *P* = 0.052) was still an independent significant risk factor (Table 5). For prediction of neurological deterioration, the most common major complication, the number of involved vertebrae (*OR* = 1.729 *P* = 0.025), lumbar location (*OR* = 9.818, *P* = 0.006) and combined approach (*OR* = 10.543, *P* = 0.029) were significant risk factors (Table 6). For those patients who had not experienced major complications, only surgical time (*OR* = 1.003, *P* = 0.029) was a significant risk factor for minor complication occurrence (Table 7).

**Table 4:** Demographic and surgical characteristics of patients receiving initial or revision surgery

Demographics	Initial (*n* = 64)	Revision (*n* = 22)	P	Total (*n* = 86)
Age (y)	36.4 ± 13.7	45.7 ± 16.6	0.010*	38.8 ± 14.9
Gender (male/female)	27/37	13/9	0.170	40/46
Body mass index (kg/m^2^)	23.7 ± 3.3	24.6 ± 4.6	0.331	23.9 ± 3.7
Surgical parameters				
*Multisegments*	23 (36%)	18 (82%)	0.000*	2.0 ± 1.2
*Surgical time (min)*	538.5 ± 265.1	617.2 ± 232.2	0.219	558.6 ± 258.1
*Total blood loss (ml)*	1415.2 ± 906.8	1854.6 ± 1313.9	0.086	1527.6 ± 1036.0
*Hospital stay (d)*	12.8 ± 10.2	17.6 ± 16.6	0.111	14.0 ± 12.2
*Major complication*	10 (16%)	8 (36%)	0.040*	0.3 ± 0.7
*Minor complication*	38 (59%)	17 (77%)	0.135	1.1 ± 1.0
Margin (*Wide/ Marginal/* Intralesional)	33/12/19	9/4/9	0.602	42/16/28

*Difference was significant at the level of 0.05

**Table 5:** Predictivity of major complications

Variable	Univariate analysis			Multivariate analysis^†^		
	OR	95%CI	P	OR	95%CI	P
Age	1.006	0.971-1.041	0.756			
Male gender	1.194	0.422-3.376	0.739			
Body mass index (kg/m^2^)	1.074	0.934-1.235	0.318			
*Neurological impairment*	0.992	0.311-3.162	0.989			
*Previous surgical history*	3.086	1.027–9.269	0.045*	2.029	0.454-9.062	0.354
*Number of involved vertebrae*	1.652	1.078–2.531	0.021*	1.445	0.800-2.612	0.222
*Lumbar location*	3.509	1.274–9.662	0.012*	3.363	0.760-14.879	0.110
*Malignancy*	1.731	0.570-5.254	0.333			
*Metastases*	1.089	0.206-5.758	0.920			
*Combined approach*	25.815	3.243–205.49	0.002*	9.236	0.977-87.303	0.052*
*Staged surgery*	6.576	1.856–23.298	0.004*	3.949	0.838-18.620	0.083
*Spondylectomy*	1.311	0.316-5.447	0.709			
*Neoadjuvant Therapy*	0.806	0.279 − 2.330	0.691			
*Preoperative embolization*	1.077	0.310-3.738	0.907			
*Surgical time (min)*	1.002	1.000-1.004	0.038			
*Total blood loss (ml)*	1.001	1.000-1.001	0.003*			

*Regression was significant at the level of 0.05; ^†^ Hosmer–Lemeshow test = 0.164

**Table 6:** Predictivity of neurological deterioration

Variable	OR	95%CI	P
*Previous treatment*	1.286	0.302-5.474	0.734
*Number of involved vertebrae*	1.729	1.076–2.985	0.025*
*Lumbar location*	9.818	1.930-49.953	0.006*
*Combined approach*	10.543	1.272–87.365	0.029*
*Staged surgery*	2.829	0.627-12.768	0.176

*Regression was significant at the level of 0.05

**Table 7:** Predictivity of minor complications in patients who had not experienced major complications

Variable	OR	95%CI	P
Age	1.009	0.976-1.044	0.585
Male gender	1.278	0.468-3.489	0.632
Body mass index (kg/m2)	1.055	0.912-1.221	0.471
*Neurological impairment*	1.773	0.549-5.723	0.338
*Previous surgical history*	2.333	0.582-9.358	0.232
*Number of involved vertebrae*	1.557	0.887-2.732	0.123
*Lumbar location*	1.258	0.407-3.888	0.690
*Malignancy*	1.970	0.629-6.166	0.244
*Metastases*	0.181	0.032-1.018	0.052
*Combined approach*	2.739	0.914-8.205	0.072
*Staged surgery*	2.949	0.324-26.826	0.337
*Spondylectomy*	0.641	0.155-2.655	0.540
*Neoadjuvant Therapy*	0.898	0.330-2.442	0.833
*Preoperative embolization*	0.533	0.151-1.884	0.329
*Surgical time (min)*	1.003	1.000-1.005	0.029*
*Total blood loss (ml)*	1.001	1.000-1.001	0.156

*Regression was significant at the level of 0.05

## Discussion

By enabling surgery to achieve marginal to wide margins, en bloc resection applied the radical oncosurgical concepts of compartment-orientated resections to the spine [11–13]. Treatment of spinal aggressive benign and malignant tumors with en bloc resection under Weinstein-Boriani-Biagini system was beneficial in terms of better local control and prognosis [14]. However, it is highly demanding and risky because of the need for extensive excision and dissection, and more challenging in thoracic and lumbar spine that require vertebral resection and reconstruction. The outcomes informing the practice remained relatively unclear because the data guiding practices were the results of limited heterogeneous case series of varying quality, using different techniques across multiple locations for a range of pathologies [2, 3, 14–16]. Therefore, we summarized the prospectively collected data of 86 consecutive patients who underwent en bloc resection under the guidance of the Weinstein-Boriani-Biagini system and anterior column reconstruction for thoracic and lumbar tumors at our institution. We tried to answer the question of whether it is safe and feasible in terms of perioperative complications and mortality.

In this study, en bloc resection was performed in all patients as planned, including tumors located from the cervicothoracic to the lower lumbar spine, recurrent tumors involving six levels, and tumors with large paravertebral masses compressing blood vessels and lungs. A wide or marginal margin was achieved in 58 patients (67.4%). 72.1% of the patients experienced complications, and 20.9% of them had major complications, which substantially altered an otherwise full and expected course of recovery.

According to Demura and his collogues [5], hardware failure is a common complication, with a perioperative incidence rate of 26.7%, of which 11.1% require surgical intervention. Compared to it, the current data shows great improvement. Among 86 objects, only 2 needed revision surgery due to hardware failure during the follow-up, which may demonstrate the advantages of 3D-printed artificial vertebral body in spinal reconstruction. To be specific, it can be attributed to three aspects: (1) 3D-printed artificial vertebral body increases the contact area and support, (2) matches the local anatomical structure and (3) provides reliable fixation effect. Therefore, 3D-printed artificial vertebral body is superior in immediate postoperative stability.

3D-printed artificial vertebral body have a larger volume than titanium mesh. Conventionally, it may suggest that surrounding structures are prone to damage during placement. However, the current data indicates that compared to titanium mesh, there was no increase in complications of vascular, visceral, or nerve injuries caused by 3D-printed artificial vertebral body.

Predictors of major complications included combined approach, staged surgery, number of involved vertebrae and lumbar location. Combined approach remained an independent risk factor in the multivariate analysis. Which means that the risk of major complications increases when a tumor breaks through the anterior cortex of the vertebral body, or the tumor is large or located in the lower lumbar spine and needs to be removed anteriorly. In addition, a previous surgical history will further increase the risk. We also found that there was no significant difference in the incidence of major complications for tumors suitable for en bloc resection, whether primary malignant, benign aggressive, or solitary metastases, regardless of whether preoperative neoadjuvant therapy was performed. Predictors of the most common (10/18) major complication, neurological deterioration, were the number of involved vertebrae, combined approach and lumbar location, which could be explained by the combined approach requiring more extensive excision and dissection, greater manipulation of the spinal cord and its blood supply, and an impact on lumbar nerve root function.

When a patient did not experience a major complication through the whole recovery, we wondered if there was a way to predict and avoid the occurrence of minor complications. However, apart from the surgical time, which could not be well controlled by the surgeon, we found nothing. Specifically, there were 24 (27.9%) dural tears and 35 (40.7%) pleural ruptures in our records, and we had not yet found an effective way to prevent these injuries.

In the spine, different tumors have different behaviors and prognosis. However, the biological behavior of tumor cells has no significant impact on perioperative complications. Indicators of effectiveness of en bloc resection, such as the long-term follow-up observations of fixation failure, local recurrence, and survival, were also beyond the scope of this study.

There are also some limitations in this article. First of all, due to the design of a retrospective study, selection bias was inevitable. Second, considering the heterogeneity of patients, the similar surgical procedures of en-bloc resections but different resected segments, operative methods, and pathological subtypes would all affect the final conclusion. Therefore, we plan to implement prospective research in the future to better reduce deviations in the process of research design and implementation. Third, the frequency of instrumentation failure and revision surgery generally increase when patient’s prognosis and postoperative follow-up is longer. The longer-term observation of internal hardware failure will be further investigated in the future research.

This study presented a comprehensive exploration of perioperative clinical results of en bloc resection and anterior column reconstruction for thoracic and lumbar spinal tumors. This procedure is associated with a high risk of complications, especially when a combined approach is needed. However, it can be performed safely in experienced hands with the help of multidisciplinary team.

## Data Availability

Data is available on request to the corresponding author.
